# Hyperproduction of nattokinase from *Bacillus subtilis* VIT MS2 using random mutagenesis and statistical optimization through central composite design

**DOI:** 10.1186/s12866-025-04150-w

**Published:** 2025-07-08

**Authors:** Merlyn Keziah S., Mohanasrinivasan V., Maneesha M., Subathra Devi C.

**Affiliations:** Department of Biotechnology, School of Bio Sciences and Technology, Vellore Institute of Technology, Vellore, Tamil Nadu 632014 India

**Keywords:** Thrombosis, Fermented foods, *Bacillus subtilis*, Mutation, Agro-rsidues, Myocardial infarction

## Abstract

**Background:**

An increase in worldwide death rates attributed to ischemic stroke and myocardial infarction explains the demand to search for new thrombolytic drugs. The current study investigates the therapeutic effect of Nattokinase, a fibrinolytic protein from the mutant strain *Bacillus subtilis* VITMS 2 isolated from fermented milk of *Vigna unguiculata*.

**Results:**

The enzyme production was improved using random mutagenesis combined with statistical optimization through composite central design (CCD) with agro-residual substrates. Among all the different combinations employed, 10% *(v/v)* cane molasses, 12.5 g/L soybean waste, 12.5 g/L eggshell powder, and 27.5 g/L brewer’s spent grain demonstrated a significant influence on fibrinolytic enzyme yield was 4639.43 ± 10.65 FU/mL (Fibrinolytic units/milliliter). This represents a ~ 37.75-fold increase when compared to the unoptimized wild-type strain. The CCD model demonstrated high significance (*p* < 0.0001) with a strong correlation (R^2^ = 0.9963), indicating a reliable model fit. The identity and purity of the enzyme was confirmed via MALDI-TOF.

**Conclusion:**

The combination of strain improvement through mutagenesis and media optimization enhanced Nattokinase production, offering a promising approach to develop an enzyme therapy for ischemic stroke and myocardial infarction.

## Introduction

Thrombosis is the pathological formation of a blood clot (thrombus) within a blood vessel, often triggered by vascular injury, endothelial dysfunction, or hypercoagulability [1]. The thrombus consists primarily of a fibrin mesh entangling platelets and red blood cells, which can obstruct blood flow and lead to serious clinical outcomes such as myocardial infarction, stroke, and pulmonary embolism. Thromboembolic diseases are accounted for one quarter of global mortality rate [2]. According to data from the Global Burden of Disease, Injuries, and Risk Factors Study (GBD) 2019, the global burden of cardiovascular diseases (CVDs) has increased substantially between 1990 and 2019. During this period, the total number of CVD cases rose from 272 million to 523 million, CVD-related deaths increased from 12.1 million to 18.6 million, and disability-adjusted life years (DALYs) attributable to CVDs grew from 279.8 million to 393.1 million, reflecting a significant rise in both prevalence and impact across populations [3].

Thrombolytic enzymes are serine proteases that act on thrombus; to disintegrate it into fibrin degraded products, this process is called fibrinolysis or thrombolysis. This occurs naturally in our body by the action of innate thrombolytic or fibrinolytic enzymes such as plasmin, urokinase and tissue plasminogen activators. The action of innate fibrinolytic enzymes becomes insufficient where there is a hemostatic imbalance. Streptokinase, staphylokinase, actinokinase, nattokinase and serrapeptidase are some of the well-known microbial fibrinolytic agents. Unlike other fibrinolytic enzymes, Nattokinase lacks side effects. Nattokinase (NK) is a serine protease enzyme obtained from natto; a traditional Japanese food produced from the fermented soybean with a type of bacteria called *Bacillus subtilis* where the enzyme plays a key role in protein degradation during fermentation [4]. *Bacillus subtilis* is a Gram-positive, rod-shaped, endospore-forming bacterium that is extensively studied and used to produce a wide array of industrial enzymes [5]. Its safety, genetic tractability, and efficient protein secretion have established it as an important organism in microbial biotechnology [6]. Due to its Generally Recognized as Safe (GRAS) status and its natural presence in fermented foods, *B. subtilis* serves as a favorable host for producing bioactive compounds, including therapeutic enzymes like Nattokinase, which have gained attention for cardiovascular disease management [7, 8].

Fermented foods serve as natural sources of proteolytic enzyme producing microbes. Even now, it is common in Japan to eat natto as breakfast. This was most valued since it was believed to have several health benefits. *Natto* has nearly double the amount of calcium and much vitamin E than boiled beans. Thus, *Natto* stands out to be an exemplary food with eighteen valuable amino acids and an enzyme *natto*kinase, the essential Clot-buster [9]. Studies reported that *Bacillus sp.* and *Pseudomonas sp.* as well as marine creatures tend to produce NK [10–12]. Nattokinase can dissolve blood clots by directly breaking down fibrin and plasmin substrate. It converts prourokinase to urokinase, degrades plasminogen activator inhibitor-1, and enhances tissue plasminogen activator, which promotes fibrinolytic action [7]. Apart from fibrinolytic activity, NK possess antithrombotic, anti-inflammatory, and antioxidant effects [8]. Epidemiological reports state that Natto consumption could prevent osteoporosis [13]. In a study conducted among the residents of Takayama, Japan [14]. The dietary intake of soy protein (Natto) has significantly reduced the number of incidences of ischemic heart diseases and ischemic strokes, even among people with familial history. It is also used in the treatment of f hypertension, Alzheimer’s disease, and vitreoretinal disorders. NK can enhance cognitive performance, lessen inflammation in the brain, and relieve conditions associated with blood brain barrier malfunction [7, 15]. However, the production of NK can be limited by the strain’s natural enzyme yield. To address this, the current study focuses on improving NK production by subjecting *Bacillus subtilis* VITMS 2, isolated from fermented cowpea milk [16], to mutagenesis and subsequent statistical optimization. These approaches aim to enhance the strain’s fibrinolytic enzyme production, which may contribute to the development of more effective NK-based therapies for cardiovascular diseases.

## Materials and methods

### Strain maintenance

Classical strain improvement methods have been carried out to improve the microbial performance. The wild -type *B. subtilis* VITMS2 (NCBI Accession no: MK156734) was maintained in tryptic soy agar and precultured in tryptic soy broth with the optimized parameters such as 4.0% inoculum, with 1% (*w/v*) sucrose, 2% (*w/v*) soya bean meal and 2% (*w/v*) malt extract and 10 mM of CaCl_2,_ MgSO_4,_ Na_2_HPO_4_ and K_2_HPO_4_ for 48 h with pH7.0 at 30℃ [17]. The culture was maintained in the same media and sub-cultured every week.

### Physical mutagenesis by UV-irradiation

The wild-type *Bacillus subtilis* VITMS 2 strain was cultured for a period of 12 h until it reached the absorbance of 0.6 at 600 nm.The culture was centrifuged at 8000 rpm for 10 min, and the pellets obtained were resuspended in phosphate buffer saline (pH 7). The spore suspension of *B. subtilis* VITMS2 (1 × 10^7^ spores/mL) maintained in 50 mM of phosphate buffered saline was poured in a sterile petri-dish placed at a distance of 20 cm away from the UV lamp (265 nm). The culture was exposed for 90 min). About 1.0 mL of the sample is withdrawn at regular intervals, transferred to amber-colored tubes, and stored in a dark chamber to avoid photo-reactivation. It was then serially diluted up to10^−8^ and plated onto fibrin agarose plate. The survival rate of the mutant strains was determined after 24 h of incubation by colonies and intensity of zones formed [18]. The survival curves were analyzed by Kaplan-Meier plot method. The colonies obtained and survival percentage was calculated using the formula: Survival (%) = (No. of colonies/mL after treatment/No. of colonies/mL in untreated control) × 100. The kill percentage was calculated as Kill (%) = 100 - Survival (%). Log survival values were calculated using the formula Log survival = Log (No. of colonies/mL/initial sample volume). Survival and kill % were determined to optimize mutagenesis conditions, ensuring sufficient mutation frequency, and were subsequently correlated with enhanced fibrinolytic enzyme activity to select high-producing NK mutants.

### Chemical mutagenesis by mutation

The UV-treated isolates with a death rate of 85% were tested for mutagenicity through Ames test [19] with slight modifications. In brief, about 0.8% (w/v) of soft nutrient agar was prepared. The cell suspensions were treated with variable concentrations up to 50 µg/mL of ethyl methanesulfonate (EMS) solution at 37 °C for 30 min. After mutagenic treatment the cells were pelleted down at 8000 rpm for 10 min and washed twice with 50 mM phosphate buffer (pH 7) saline. The suspensions are then serially diluted and plated onto nutrient agar plates. The mutants were screened for partial clot lysis activity [1]. The putative positives were confirmed with fibrin degradation assay [20]. Serial dilutions of EMS-treated samples were plated, and colonies were counted after 24 h. The survival and kill percentages were calculated in the same manner as for the UV treatment, using the control group (no EMS) as the baseline. The survival curves were analyzed by the Kaplan-Meier plot method. The log survival was calculated in the same way as for UV treatment.

### Statistical optimization of production parameters through central composite design

After subjecting the organism to physical and chemical mutagenic treatments, the potent mutant strain was considered for screening of organic substrates for the fibrinolytic protease production. The combination of statistical and mathematical techniques minimizes random noise and stochastically obtains maximum results with fewer experimental trials. To plot the response using regression analysis, the key parameters were assessed. In order to enhance production, agro-residues were used in statistical optimization to replace the carbon and nitrogen sources. Since calcium chloride had a beneficial effect on the development of enzymes, it was replaced by eggshell powder; soy bean was replaced by soybean waste and another nitrogen source, brewer’s spent medium; and sugar cane molasses was used in place of sucrose [21–24]. Based on the one-factor-at- a time (OFAT) experimental result [25], the factors such as incubation period and inoculum size were kept constant at an optimal level. The optimized factors were elucidated using response surface methodology (RSM). A five-level-four factor central composite design (CCD) of RSM was constructed considering sugar cane molasses (SCM) (Dhampur green, FMCG manufacturers, Delhi), de-oiled soybean waste (SBW) (Coastline Eximpex, Bangalore, Karnataka), eggshell powder (ESP) (KK eggshell powder, Trichy, Tamil Nadu) and brewer’s spent grain (BSG) (Mandi Mohalia, Mysuru, Karnataka) as substrates to identify their optimal concentrations. The essential factors are optimized with the central composite design, and a matrix of 30 experimental combinations were randomly chosen and run by the software Design Expert 12.0 (Stat-Ease, Inc., Minneapolis, MN, USA). The extracellular protease of the mutagen-treated *B. subtilis* VITMS 2 was produced using CCD-optimized media under controlled conditions, which was optimized through the OFAT method. The enzyme was purified and examined for fibrinolytic activity and protein content [26].

### Protein determination

Lowry’s method was used to estimate the protein concentration with bovine serum albumin as the standard [27].

### Determination of fibrinolytic activity

The fibrin degradation ability of the enzyme was measured through detection of tyrosine liberation [25]. The reaction mixture consisted of 100 µL of enzyme solution, pre-incubated with 300 µL of 100 mM Tris-HCl buffer (pH 7.4) containing 10 mM CaCl₂ for 5 min at room temperature. About 300 µL of 1.2% (w/v) fibrin solution was added, and the mixture was incubated at 37 °C for 10 min. The reaction was terminated by adding 600 µL of chilled trichloroacetic acid (TCA) reagent to precipitate the proteins. The liberated tyrosine in the supernatant was then measured at 275 nm using a UV spectrophotometer (UVmini-1240, SHIMADZU). A standard tyrosine calibration curve was used to calculate the enzyme activity. One fibrinolytic unit (FU) was defined as the amount of enzyme required to release 1 µg of tyrosine per minute under assay conditions. A standard tyrosine solution was used as the positive control, while a blank reaction without enzyme served as the negative control.

### Partial clot lysis

Partial clot lysis of NK was determined using a modified method [28]. A plasma clot is prepared in a glass tube using 0.7 mL of 100 mM HEPES buffer (pH- 7.4), 0.1mL red blood cell suspension, 0.1 mL of 75 U/mL thrombin, 0.1 mL of 1% (*w/v*) reconstituted fibrinogen. 100 µL of crude samples were added to the prepared clot. The clot lysis is detected with spectrophotometric detection of RBC release at 542 nm. The percentage of red blood cells released within 20 min corresponds to the percentage of clot lysis. Streptokinase was used as the positive control, and untreated clots (without enzyme) served as the negative control.

### Protein purification

The extracellular crude was preceded for saturation of proteins using ammonium sulphate. Salt precipitation is preferably used because of the high-water solubility of ammonium sulphate and that it follows the Hofmeister series that enables better protein stability [29]. The supernatant was kept in a cold bath throughout the procedure, and ammonium sulphate salt was added slowly with a gentle stir to prevent the formation of clumps. The saturation gradient ranges from 20 to 80% [30]. After the dissolution of salt, the media was centrifuged at 10,000 rpm for 20 min at 4 °C (REMI C-24 BL) to get a pellet. The pellet formed is resuspended in 10mM Tris HCl buffer pH7.4. The desalted enzyme solution was applied onto 15 cm x 5 mm column with a bed volume of 3.0 mL anionic exchanger DEAE Sepharose FF resin (GE Healthcare Bio- Sciences, Uppsala, Sweden) dispersed in 50mM Tris-HCl containing 20mM CaCl_2_ pH 7.4. The tightly bound particles of the protease were eluted with 150-500mM NaCl. The elution was carried out at the flow rate of 0.2 mL/min. The eluates were collected and analyzed for protein content and enzyme activity [31].The protein fractionates collected was further loaded onto Sephadex G-50 resin dispersed in 50mM Tris-HCl with 20mM CaCl_2_ pH 7.4. The porous matrix Sephadex G-50 acts as a molecular sieve with a fractionation range of 3 × 10^3^ −2 × 10^4^. The purified elutes were collected in small 2.0 mL microfuge tubes and assessed for protein concentration and enzyme activity [32]. Finally, the purified fractions were concentrated using ultrafiltration with a 30 kDa molecular weight cutoff (MWCO) membrane (Amicon Ultra-15 centrifugal filter units, Merck, Darmstadt, Germany) by centrifugation at 6,000 rpm for 30 min at 4 °C. This step concentrated the purified enzyme preparation for downstream assays and SDS-PAGE analysis [33].The identification of protein produced was conducted using SDS-PAGE. The samples were loaded onto the SDS- PAGE, with 4% stacking gel and 12% separating gel [34]. The gel was run in standard SDS-PAGE apparatus, and the molecular mass of the protein was estimated based on the separation of the samples relative to a protein ladder [27].

### Matrix-assisted laser desorption ionization time-of-flight mass spectrometry

The protein band from SDS-PAGE gel was excised and digested with sequencing-grade modified trypsin (Promega) according to the modified method [35]. The excised gel was subjected to dehydration in acetonitrile and dried. The gel was then immersed in 100 mM NH_4_HCO_3_ containing 10mM dithiothreitol (DTT) and reduced at 56 °C for 60 min. Then cooled to room temperature and 55mM iodoacetamide in 100mM NH4 HCO3 was added. The gel was removed after 45 min of incubation and washed with 0.1 mL of 100 mM NH4 HCO3, subjected to dehydration on addition of acetonitrile and then rehydrated on adding 100 mM NH4 HCO3. The gel pieces were dried and resuspended in digestion buffer (50mM NH_4_ HCO_3_, 5mM CaCl2) containing trypsin in a cold-bath for 45 min. The gel pieces were removed from the buffer, replaced with same buffer without trypsin and incubated at room temperature overnight allowing the enzymatic cleavage. The peptides were extracted with 20mM NH_4_ HCO_3_. The matrix material (α-cyano-4- hydroxy-trans-cinnamic acid) and nitrocellulose (1:1 *v/v*) were dissolved in acetone. Approximately 0.5µL of the matrix-nitrocellulose solution was spread onto the stainless-steel stage. Aliquots of analyte solution (0.5 µL) were applied onto the matrix- solvent surface and allowed to evaporate. Thus, the monoisotopic masses of the digested protein were analyzed using m/z spectra (Bruker Daltonics). The purified enzyme samples were further analyzed to confirm the presence of Nattokinase by performing HPLC [36].

## Results

### Effect of UV irradiation on partial clot lysis and NK activity

**Fig. 1:**
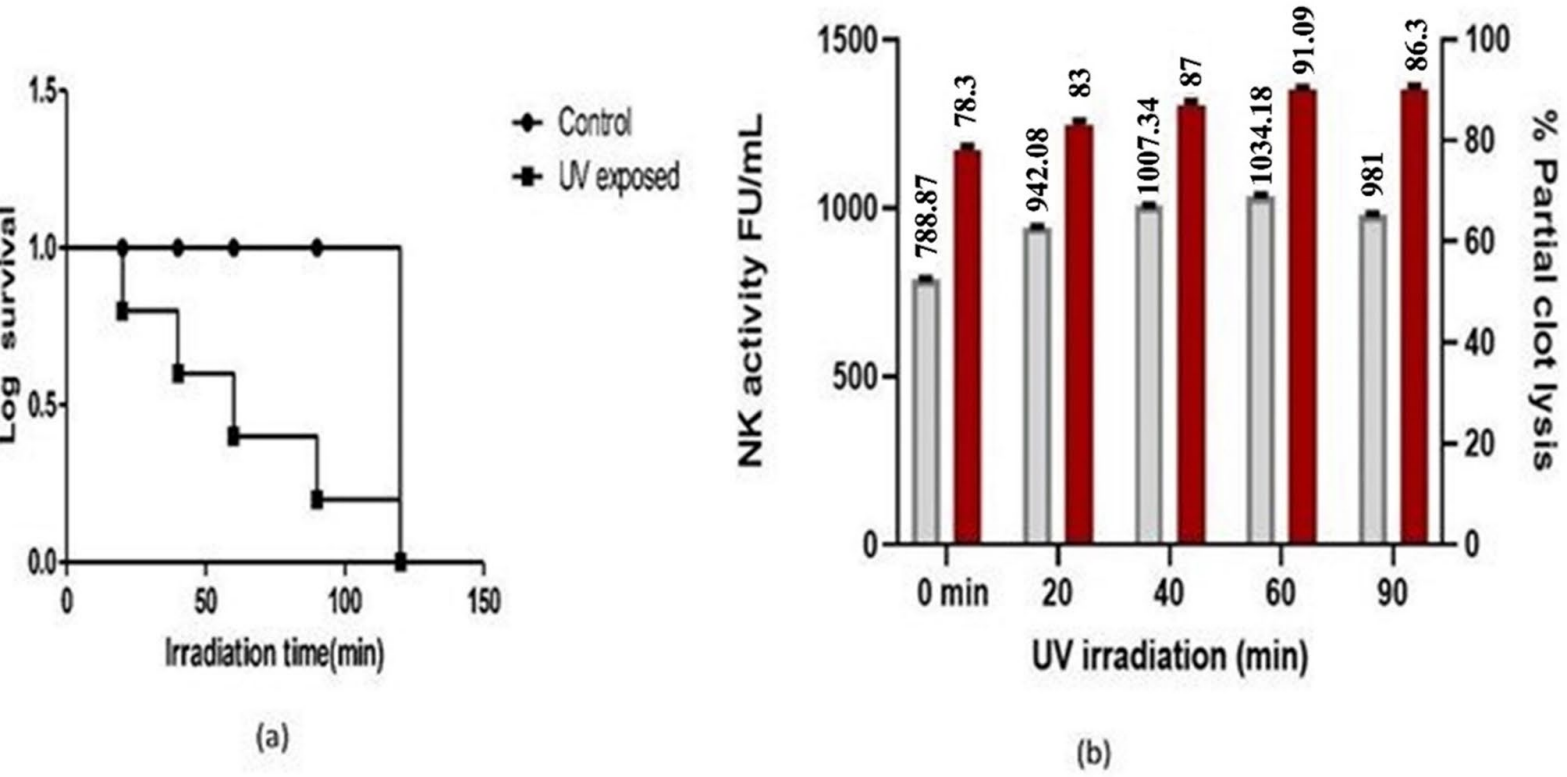
**a** Kaplan Meier plot of mutant *Bacillus subtilis* VITMS2 under UV irradiation **b** Fibrinolytic activity (FU/mL) and partial clot lysis (%) of UV-mutated *Bacillus subtilis* VITMS2 isolates. The red bar represents the % of partial clot lysis, while the grey bar represents the fibrinolytic activity measured in fibrinolytic units (FU/mL)

The wild-type parental strains were subjected to UV irradiation for different time intervals of 0, 20, 40, 60, 90, and 120 min. Increasing the exposure time decreased the survival rate of the bacteria and altered protease activity. Thirty-nine mutant strains were isolated, and they were sub-grouped according to their hydrolytic activity on fibrin agar plates. Amongst these four strains, UVBS-13, UVBS-18, UVBS-23, and UVBS-31 exhibited high clearance zones. Out of all the isolates investigated, UVBS-18 exposed to 60 min irradiation was found to have a high percentage of partial clot lysis of 93.1 ± 0.8% and fibrinolytic activity of 1034.18 93.1 ± 0.5 FU/mL (Fibrinolytic units/mL) compared to other mutant derivatives. Irradiation exposure of 60 min resulted in a 50% lethality rate. Since, UVBS-18 demonstrated the highest fibrinolytic activity, it was selected for further treatment with a chemical mutagen. The fibrinolytic activity and percentage of partial clot lysis of UVBS-31 reached 90 ± 0.8% at 90 min irradiation. The lethality rate was observed to be 91.49% with 90 min irradiation. The survival rate of *Bacillus subtilis* VITMS 2 was 33.19% after 90 min of UV exposure. The lethality rate was 100% with 120 min exposure time. The data Table 1 shows the survival and kill percentage of *B. subtilis* VITMS 2 after UV treatment. Figure 1a and b depict the survival curve and activity of NK fibrinolytic activity and partial clot lysis activity of UV mutated isolates of *Bacillus subtilis* VITMS 2. Effect of EMS mutagen on partial clot lysis and NK activity.

**Table 1:** Effect of UV irradiation on *B. subtilis* VITMS 2

Irradiation time (min)	No of colonies/mL X 10^7^	Kill (%)	Survival (%)	Log survival
Wild-type	224 ± 4.6	0	100	2.0 ± 0.73
20	188 ± 1.09	36.7 ± 0.86	63.3 ± 0.79	1.96 ± 0.6
40	117 ± 1.74	62.2 ± 0.92	37.8 ± 0.68	1.66 ± 10.48
60	44 ± 0.43	73.1 ± 1.14	26.9 ± 1.72	1.25 ± 15.36
90	6 ± 0.81	94.3 ± 0.88	5.7 ± 1.81	1.05 ± 10.36
120	-	100	-	-

### Effect of EMS mutagen on partial clot lysis and NK activity

**Fig. 2:**
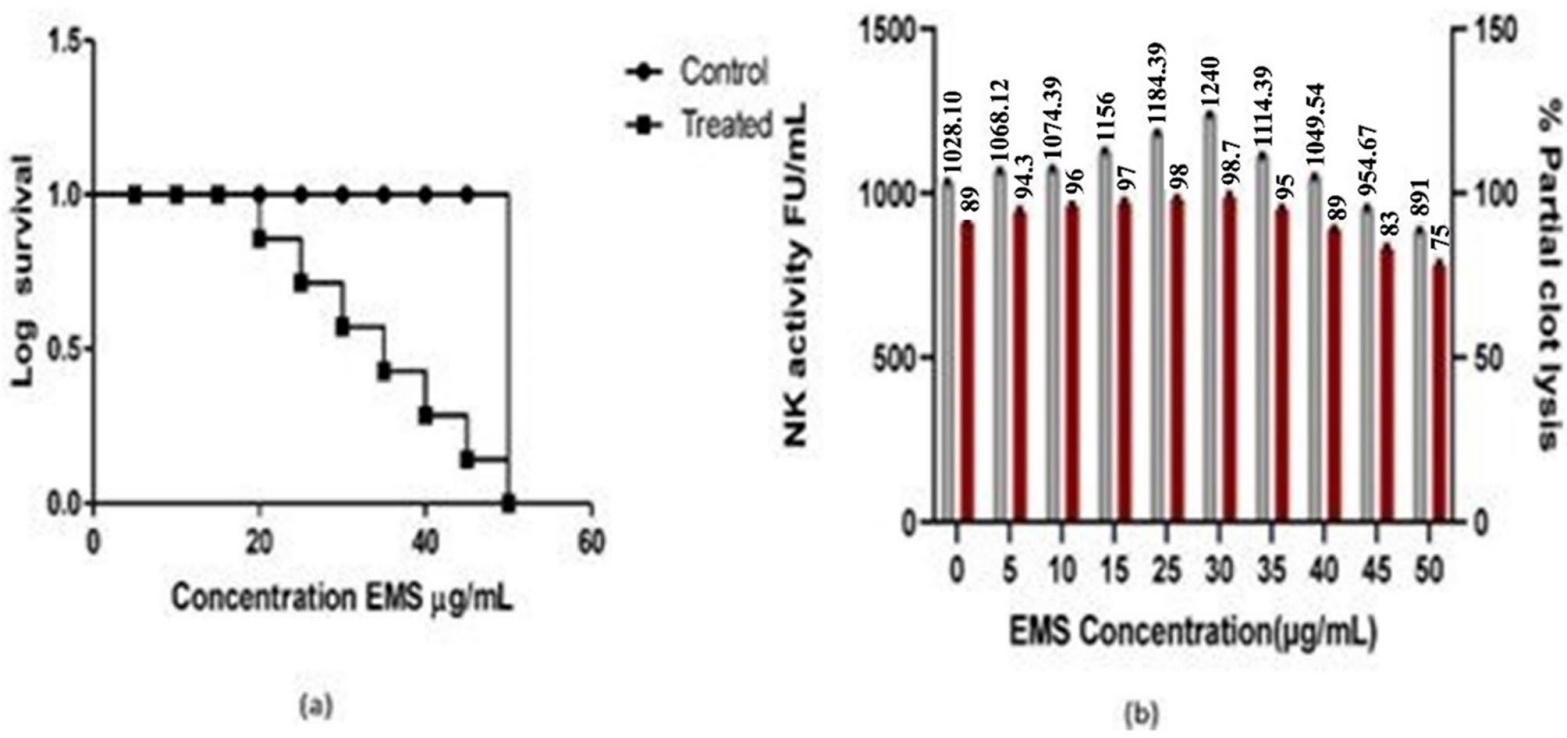
**a** Kaplan Meir plot of mutant *Bacillus subtilis* VITMS2 under EMS mutagen **b** Fibrinolytic activity of EMS-mutated *Bacillus subtilis* strains. The red bar represents the % of partial clot lysis, while the grey bar represents the fibrinolytic activity measured in fibrinolytic units (FU/mL)

The potent strains with high proteolytic zones were screened for susceptivity with chemical mutagens ethidium bromide (EtBr), ethyl methane sulfonate (EMS), and silver nitrate (AgNO_3_) using Ames’s test. The mutagen ethyl methane sulfonate (EMS) was selected as the organism was found to be highly resistant to EMS than the other two cohorts. The selected strain was subjected to different concentrations of EMS treatment from 5 to 50 µg/mL. Increasing concentrations of EMS treatment decreased the survival rate of the bacteria and altered its activity. A total of 28 isolates showed hydrolytic zone on fibrin agar plates. Out of which, EMSBS-6 and EMSBS-14, and EMBS-20 are observed to be more potent. The isolate EMSBS-14 treated with 30 µg/mL concentration of EMS exhibited high fibrinolytic activity of 1240 ± 1.09 FU/mL and 98.7 ± 1.0 partial clot lysis. This is depicted in the log survival curve which expressed a gradual decrease from 2.00 to 0.93. At 50 µg/mL concentration, the lethality rate was found to be 100%. The data Table 2 shows the survival and kill percentage of *B. subtilis* VITMS 2 after EMS treatment. Figure 2a and b depict the survival curve and activity of NK fibrinolytic activity partial clot lysis activity of EMS mutagen treated isolates of *Bacillus subtilis* VITMS 2.

**Table 2:** Effect of mutagen Ethyl methane sulfonate EMS treatment on *B. subtilis* VITMS

EMS treatment (Conc µg/mL)	No of colonies/mL X 10^7^	Kill (%)	Survival (%)	Log survival
0	228 ± 3.4	0.0	100	2.01 ± 0.17
5	186 ± 6.1	11.4 ± 3.12	88.6 ± 0.98	1.94 ± 0.86
10	171 ± 4.2	27.6 ± 2.05	72.4 ± 0.90	1.85 ± 0.31
15	160 ± 3.6	32.2 ± 1.48	67.8 ± 0.87	1.83 ± 0.42
20	147 ± 2.8	40.9 ± 1.21	59.1 ± 0.84	1.77 ± 0.80
25	131 ± 2.4	41.8 ± 1.03	47.6 ± 0.81	1.67 ± 0.6
30	120 ± 2.2	52.4 ± 0.96	38.2 ± 0.74	1.58 ± 0.91
35	93 ± 1.6	74.9 ± 0.95	25.1 ± 0.70	1.39 ± 0.07
40	49 ± 1.2	86.6 ± 0.88	13.4 ± 0.62	1.12 ± 0.05
45	21 ± 1.0	91.3 ± 0.56	8.7 ± 0.68	0.93 ± 0.02
50	-	100	**-**	-

### Media optimization of the mutant strain *B. subtilis* VITMS 2

Hyper fibrinolytic protease producing mutant strain *B. subtilis* VITMS 2 EMSBS-14 was inoculated in a fermentation medium which constituted agricultural and industrial wastes as carbon and nitrogen substitutes. These substitutes were selected based on the OFAT production parameter optimization carried out with the parent strain. The optimization of OFAT production parameters was carried out using the parent strain before applying the same conditions to the mutant strains. The selection of these parameters was based on a series of preliminary experiments that assessed various factors, including incubation time, temperature, pH, carbon, and nitrogen sources. These parameters were optimized by systematically varying one factor at a time while keeping others constant (OFAT approach) and evaluating their impact on OFAT production. The most effective conditions identified from these experiments were then applied to the mutant strains to ensure consistency in the comparative analysis. The factors which showed the strongest influence on enzyme production were substituted with their organic waste alternatives. Sucrose was substituted by cane molasses; soy peptone was substituted by soybean waste and another nitrogen brewer’s spent grain since calcium chloride had a positive influence on enzyme production; it was substituted by eggshell powder. A central composite design was developed using response surface methodology with these four factors. This method enables variation of all factors, which is a major advantage compared to the classical method. Each parameter was studied for low (-), high (+) and (0) middle values and were coded at five levels (-α, −1,0, + 1, + α) (Table 3). The experiment consisted of 30 trials, as shown in Table 4. The quadratic model was expressed by the influence of cane molasses (A), soybean waste (B), eggshell powder (C), and brewer’s spent grain (D) on fibrinolytic activity (FU/mL). Cane molasses supplies a readily available carbon source, fueling glycolysis and ATP production, which enhanced protein biosynthesis. Soybean waste and brewer’s spent grain provided organic nitrogen and peptides, which activate the protease gene expression. Eggshell powder acted as a calcium ion stabilizer, protecting nattokinase from autolysis and enhancing enzyme secretion efficiency. With the combination of these agro-residual substrates, the production of the enzyme varied from 2606.48 to 4639.43 FU/mL. The Table 4 represents the actual and predicted activity of the central composite design for selected variables. These nutritional supplements have positively correlated to an increase in the production of NK fibrinolytic protein from mutant *B. subtilis* VITMS 2. For all the experiments, the pH of 7.0 and temperature of 30 °C optimized from the OFAT method was maintained.

**Table 3:** Experimental ranges of four variables in central composite design (CCD) of response surface methodology (RSM)

Code	Independent Variables	Range coding level				
		-α	−1	0	+ 1	+ α
A (X_1_)	Cane Molasses % *(w/v)*	0	5	10	15	20
B (X_2_)	Soy bean Waste g/L	−2.5	5	12.5	20	27.5
C(X_3_)	Eggshell powder g/L	−2.5	5	12.5	20	27.5
D (X_4_)	Brewers Spent Grain g/L	−2.5	5	12.5	20	27.5

**Table 4:** Design matrix of organic agro-residual substrates for fibrinolytic enzyme production using central composite design with actual and predicted values of fibrinolytic activity of mutant *Bacillus subtilis* VITMS 2

Run	Factor 1 A: Cane Molasses %	Factor 2 B: Soybean Waste g/L	Factor 3 C: Eggshell Powder g/L	Factor 4 D: Brewer’s Spent grain g/L	Actual Value FU/mL	Predicted Value FU/mL
1	15	20	5	5	3185.03	3182.79
2	5	5	20	5	2711.35	2719.50
3	0	12.5	12.5	12.5	2697.67	2696.23
4	10	12.5	−2.5	12.5	2974.63	2977.20
5	5	5	20	20	3009.12	3006.64
6	5	5	5	5	2738.76	2711.60
7	15	20	5	20	4204.54	4211.07
8	15	20	20	20	4509.42	4531.87
9	15	5	20	20	4255.64	4266.57
10	10	12.5	12.5	12.5	4178.18	4158.31
11	10	12.5	12.5	12.5	4177.23	4158.31
12	5	20	20	20	3747.35	3728.07
13	15	5	20	5	3679.52	3671.71
14	10	12.5	12.5	−2.5	3334.9	3324.01
15	5	20	20	5	2826.07	2853.66
16	10	−2.5	12.5	12.5	2913.73	2917.48
17	10	27.5	12.5	12.5	3091.37	3077.65
18	15	5	5	5	3710.09	3744.05
19	10	12.5	12.5	12.5	4075.24	4158.31
20	15	5	5	20	4217.36	4185.06
21	10	12.5	12.5	27.5	4638.51	4639.43
22	10	12.5	12.5	12.5	4171.52	4158.31
23	5	20	5	20	3323.95	3327.04
24	10	12.5	27.5	12.5	3318.44	3305.90
25	5	20	5	5	2602.72	2606.48
26	20	12.5	12.5	12.5	4541	4532.47
27	10	12.5	12.5	12.5	4174.29	4158.31
28	10	12.5	12.5	12.5	4173.42	4158.31
29	15	20	20	5	3354.24	3349.74
30	5	5	5	20	2825.7	2844.89

### Interaction study

The interactive effects of the media components were deduced by standard analysis of variance (ANOVA), regression coefficient, F-values, and *p*-values of variables were examined and illustrated in Table 5. The model F value of 932.88 implies that the model is significant. There is only a 0.01% chance that a large F-value could occur due to noise. A *P*-value less than 0.0500 indicate that model terms are significant. In this case, A, B, C, D, AB, AC, AD, BC, BD, CA, A^2^, B^2^, C^2^, and D^2^ are significant model terms. The values greater than 0.1000 indicate that the model terms are not significant. The model exhibited strong statistical parameters with an R^2^ value of 0.9963, an Adjusted R^2^ of 0.9978, and a Predicted R^2^ of 0.9941, indicating excellent agreement between the experimental and predicted values. The pre-determined R^2^ was 0.9963, in agreement with the adjusted R^2^ of 0.9978 thus, depicts the adequacy of the model to predict the response. Adequate precision measures signal-to-noise ratio greater than 4 is desirable, and the ratio is 93.5877. The “Lack of fit F value” of 0.3516 implies that the lack of fit is insignificant relative to the pure error. There is a 92.5% chance that a lack of fit F- value this large could be noise. Non-significant lack of fit is good as it confirms that the model equation was adequate to predict the enzyme yield. The value of the coefficient of variation (CV% =0.8083) revealed the precision and reliability of this model. Among all the different combinations employed, 10% *(v/v)* cane molasses, 12.5 g/L soybean waste, 12.5 g/L eggshell powder, and 27.5 g/L brewer’s spent grain when cultured in an orbital shaker of 200 rpm with a pH of 7.0 at 30 °C demonstrated a significant influence on fibrinolytic enzyme yielded a production of 4639.43 ± 10.65 FU/mL.

**Table 5:** Variance and regression ANOVA for quadratic model

Source	Sum of Squares	Df	Mean Square	F-value	*p*-value	
Model	1.233E + 07	14	8.804E + 05	932.88	< 0.0001	Significant
A-Cane Molasses	5.058E + 06	1	5.058E + 06	5359.27	< 0.0001	
B-Soybean Waste	38484.85	1	38484.85	40.78	< 0.0001	
C-Eggshell Powder	1.621E + 05	1	1.621E + 05	171.73	< 0.0001	
D-Brewer’s Spent Grain	2.595E + 06	1	2.595E + 06	2750.25	< 0.0001	
AB	2.081E + 05	1	2.081E + 05	220.46	< 0.0001	
AC	6438.46	1	6438.46	6.82	0.0196	
AD	94688.52	1	94688.52	100.33	< 0.0001	
BC	57254.92	1	57254.92	60.67	< 0.0001	
BD	3.449E + 05	1	3.449E + 05	365.46	< 0.0001	
CD	23669.82	1	23669.82	25.08	0.0002	
A^2^	5.073E + 05	1	5.073E + 05	537.50	< 0.0001	
B^2^	2.310E + 06	1	2.310E + 06	2447.44	< 0.0001	
C^2^	1.772E + 06	1	1.772E + 06	1877.91	< 0.0001	
D^2^	53460.35	1	53460.35	56.65	< 0.0001	
Residual	14155.96	15	943.73			
Lack of Fit	5844.37	10	584.44	0.3516	0.9250	not significant
Pure Error	8311.58	5	1662.32			
Cor Total	1.234E + 07	29				
R^2^						0.9963
Adjusted R^2^						0.9978
Predicted R^2^						0.9941

The final equation in terms of actual factors is written as Fibrinolytic activity FU/mL = + 799.92944 + 219.66000 A + 118.80054 B + 107.43904 C + 1.78087 D −3.04090 A * B −0.534933 A * C + 2.05143 A * D + 1.06347 B * C + 2.61011 B * D + 0.683778 C* D −5.43963 A^2^ −5.15888 B² −4.51895 C^2^−0.784859 D^2^.

### Interaction of variables

**Fig. 3:**
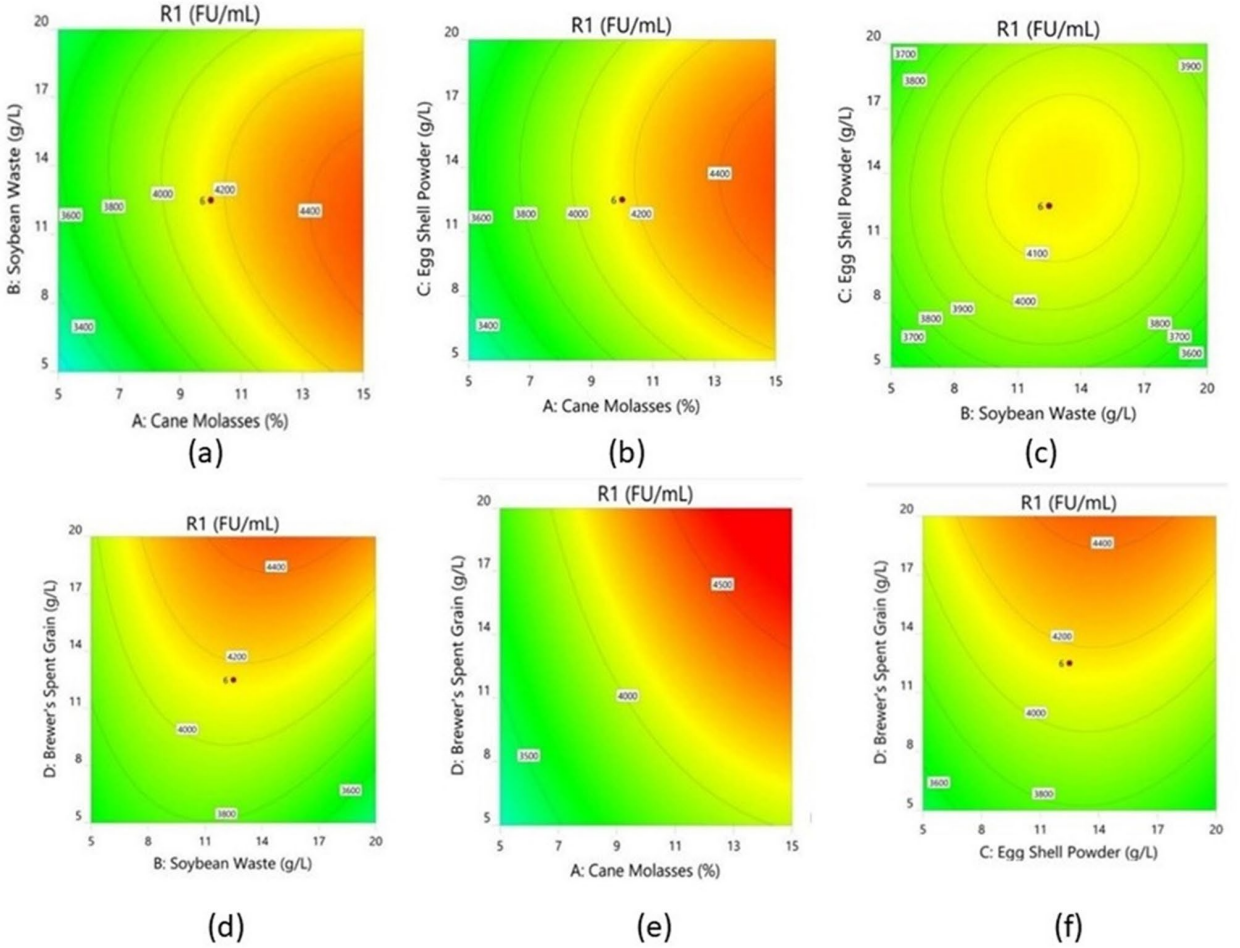
Contour plots depicting the optimization of agro-residual substrates for Nattokinase production

**Fig. 4:**
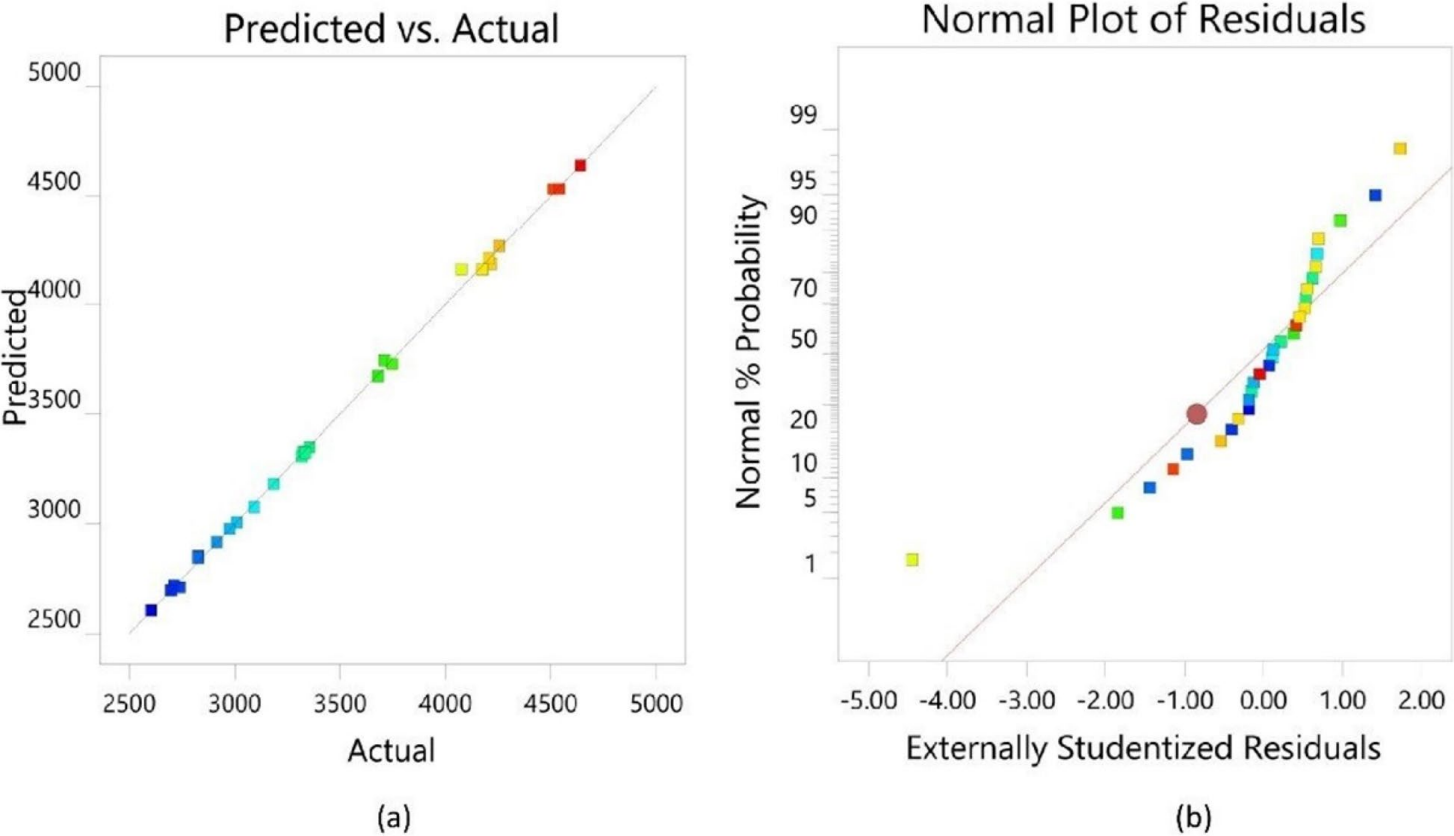
**a** Predicted vs. Actual plot for Nattokinase production optimization. **b** Normal plot of residuals for Nattokinase production data

**Fig. 5:**
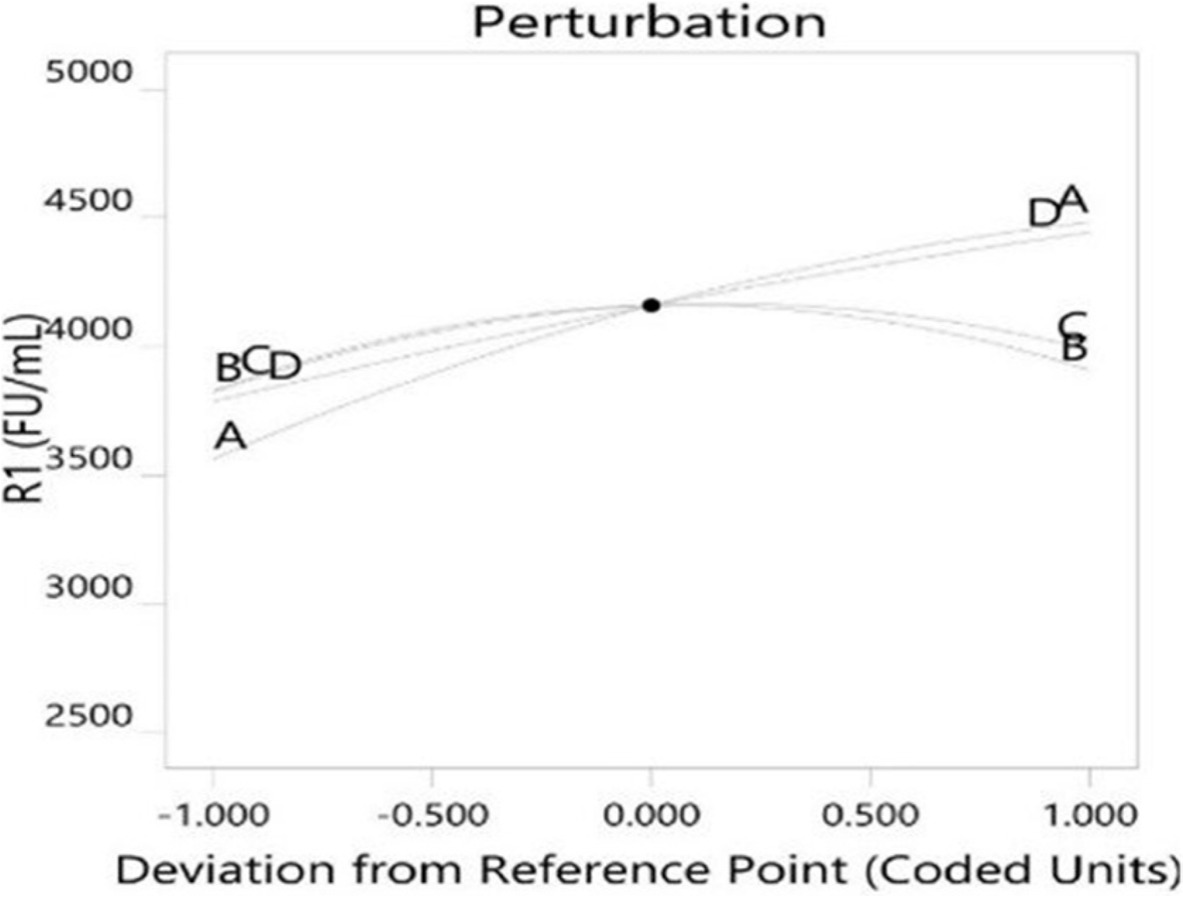
Perturbation plot showing the effect of selected variables on the Nattokinase response

Graphical representation of contour plots (Fig. 3) indicates the influence on enzyme activity. When the concentration of substrate increased, the enzyme yield was also considerably increased but further increase in concentration resulted in the reduction of enzyme yield. Increased concentration of cane molasses influences a significant increase in the production of the enzyme. An increase in soybean waste concentration considerably increases the yield of the enzyme but the effect of molasses, and eggshell powder has a greater significance on enzyme yield. The interaction between cane molasses and soybean meal; cane molasses and eggshell powder; cane molasses and brewer’s spent grain; soybean meal and brewer’s spent grain and eggshell powder and brewer’s spent grain has shown elliptical contour plots. However, it’s important to note that significance isn’t determined by the shape of the plots. Instead, the significance of these interactions comes from the regression analysis and p-values, which show that these interactions are statistically significant. The interactiom between soybean meal and eggshell powder has resulted in circular contour plots, which suggests insignificant interaction. Contour plot representing the interaction effect of (g) Soybean waste and (h) Brewer’s spent grain. Contour plot representing the interaction effect of (i) Soybean waste and (j) Brewer’s spent grain. Adequacy of the model is analyzed through a strong correlation demonstrating the precision and accuracy of the central composite design. The normal probability and correlations between actual vs. predicted through diagnostic plots (Fig. 4a and b). The statistical optimization thus gives the right amount for a combination of factors that satisfies optimal production. The positive shift of the NK enzyme production with respect to the agro-residual substrates were illustrated using an intersecting perturbation plot in Fig. 5. Table 6 represents the coefficients in terms of coded factors. Figure 5 shows that NK production is positively influenced by increasing cane molasses (A), as indicated by the upward slope and a positive coefficient (+ 459.06) in Table 6. Soybean waste (B) and eggshell powder (C) show mild positive effects, while brewer’s spent grain (D) has a negative impact, as seen by its downward slope and negative coefficient (–329.97). Interaction terms reveal that some combinations (e.g., AB, AD) negatively affect NK production, whereas others (e.g., BC, BD) are synergistic. Overall, the perturbation plot and coefficients collectively guide substrate optimization to maximize NK yield.

**Table 6:** Representation of coefficients in terms of coded factors

Factor	Coefficient Estimate	df	Standard Error	95% CI Low	95% CI High	VIF
Intercept	4158.31	1	12.54	4131.58	4185.04	
A-Cane Molasses	459.06	1	6.27	445.70	472.43	1.0000
B-Soybean Waste	40.04	1	6.27	26.68	53.41	1.0000
C-Eggshell Powder	82.17	1	6.27	68.81	95.54	1.0000
D-Brewer’s Spent Grain	328.85	1	6.27	315.49	342.22	1.0000
AB	−114.03	1	7.68	−130.40	−97.66	1.0000
AC	−20.06	1	7.68	−36.43	−3.69	1.0000
AD	76.93	1	7.68	60.56	93.30	1.0000
BC	59.82	1	7.68	43.45	76.19	1.0000
BD	146.82	1	7.68	130.45	163.19	1.0000
CD	38.46	1	7.68	22.09	54.83	1.0000
A²	−135.99	1	5.87	−148.49	−123.49	1.05
B²	−290.19	1	5.87	−302.69	−277.68	1.05
C²	−254.19	1	5.87	−266.69	−241.69	1.05
D²	−44.15 1	1	5.87	−56.65	−31.65	1.05

### Confirmation of purity and homogeneity by RP-HPLC and MALDI-TOF

**Fig. 6:**
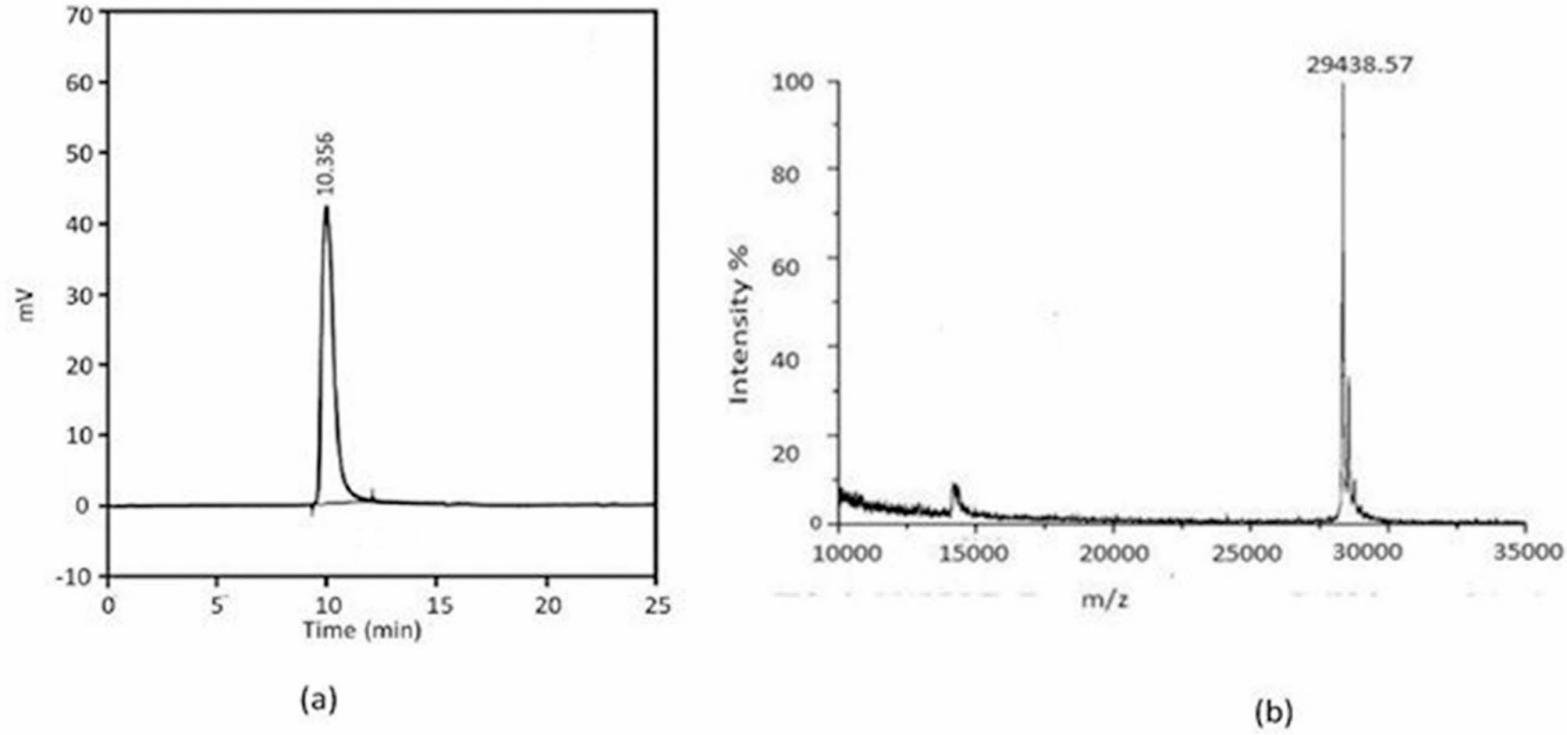
**a** RP-HPLC chromatogram of purified nattokinase **b** MALDI-TOF spectrum of purified nattokinase

The purity and homogeneity of the nattokinase enzyme were confirmed by RP-HPLC and MALDI-TOF analysis. The HPLC chromatogram exhibited a single peak with a retention time of 10.356 min, confirming the homogeneity of the purified protease, shown in (Fig. 6a). The absence of multiple peaks confirms the elimination of contaminating proteins, further validating the purity of the NK enzyme. The sharp and well-defined peak suggests a high degree of homogeneity in the sample. The MALDI- TOF analysis of purified NK protein from *Bacillus subtilis* VITMS 2 generated a single spectrum of the peak with respect to the m/z ratio. The automated analysis of spectra (Fig. 6b), signifying the presence of a single protein species. The absence of additional peaks further supports the homogeneity and purity of the enzyme preparation. SDS-PAGE analysis of the purified protein was previously published [19] and confirmed the molecular weight consistency with MALDI-TOF data. These results collectively confirm the successful purification of NK, making it suitable for further biochemical and functional characterization.

### Validation of NK protein from mutated *B. subtilis* VITMS 2

The strain improvement of mutagen treated *Bacillus subtilis* VITMS 2 cultured in the optimized media was initially determined using azocasein degradation assay. On purification, after mutagen treatment *Bacillus subtilis* VITMS 2 produced a 40.6% yield and a 148.64-fold increase in purity of the NK fibrinolytic protein. The total activity of 65,856 U with the total protein content of 106.06 mg. The active NK protein fractions were collected from Sephadex G −50 and pooled. Purified protein had a maximum of 40.6% yield and 148.64-fold purity. The purification table depicts the activity, specific activity, fold purification, yield and relative activity at each stage of purification (Table 7). On performing fibrin degradation assay, the fibrinolytic activity of purified NK protein was found to be 25,977.96 ± 5.86 FU. The specific activity was found to be 86,593.32 ± 3.44 FU/mg. Table 8 represents the fold increase of fibrinolytic enzyme activity at each stage of strain improvement and optimization. The wild-type *Bacillus subtilis* VITMS2 initially produced 122.9 ± 0.06 FU/mL. After UV and EMS mutagenesis, the selected mutant EMSBS-14 showed enhanced activity (1240 ± 1.09 FU/mL), which further increased to 4639.43 ± 10.65 FU/mL after CCD-based optimization. Fold increases were calculated relative to wild-type activity.

**Table 7:** Purification table of the production optimized mutant strain *Bacillus subtilis* VITMS2

Purification	Total protein (mg)	Relative activity U/mL	Total Activity (U)	Specific Activity (U/mg)	Fold purification	Yield %
Crude	106.06	424.87	65,856.00	621.28	1	100
Ammonium sulfate	38.95	1818.49	52,736.41	1353.95	2.719	80.07
DEAE FF	4.65	5150.54	49,445.26	10,633.37	17.11	75.08
Sephadex G-50	1.12	4840.72	38,725.82	34, 576.62	55.94	58.80
Ultrafiltration 30 MCW	0.29	4782.39	26,7 81.41	92,349.68	148.64	40.6

**Table 8:** Fold increase in fibrinolytic enzyme activity during strain improvement and CCD optimization

SI.No	Stage	Enzyme activity (U/mL)	Fold
1	Wild strain (*Bacillus subtilis* VITMS2) [25]	122.9 ± 0.06	1.0
2	After UV + EMS Mutagenesis (Mutant EMSBS-14)	1240 ± 1.09	10.09
3	After CCD Optimization (Mutant EMSBS-14)	4639.43 ± 10.65	37.75

## Discussion

Nattokinase is well known for its strong fibrinolytic and thrombolytic properties, which make it an invaluable tool in the fight against cardiovascular conditions including high blood pressure and heart disease. It is additionally recognized as an efficient, safe, and reasonably priced medication because of its therapeutic potential in treating ailments including Alzheimer’s disease, chronic pain, and uterine fibroids [37, 38]. Methods such as mutation, genetic engineering, fermentation optimization process could enhance the microbial Nattokinase production [39]. Previous study showed that the wild-strain *Bacillus subtilis* VITMS2 produced 122.9 ± 0.06 U/mL of Nattokinase enzyme [25]. This study aims to enhance the production of Nattokinase from *Bacillus subtilis* VITMS2, an isolate from the fermented milk of *Vigna unguiculata* through physical and chemical mutation and to further increase the enzyme production via optimizing the production parameters by employing RSM. Application of random mutagenesis by UV, EtBr, EMS, MMS, FU and NTG have proved to increase the protease productivity. The alkylating mutagenic EMS (ethyl methane sulfonate) cause random mutations like nucleotide substitution, point mutation, frame shifts and chromosomal aberrations. It is most prominently used for over production of amylases, proteases, lipases, cellulases, pectinases, phytases and laccases [40]. Wild-type strains *B. subtilis* A, *B. subtilis*,* B. thuringiensis* and *B. licheniformis* on EMS exposure have shown increased fibrinolytic activity [41]. A study showed that UV mutated *Bacillus sp* produced 490 µg/mL of Nattokinase [36]. Similarly, the NK activity of the mutated *Pseudomonsa aeruginosa* UV60 was found to be 4263 U mL^−1^ [42]. Furthermore, the NK yield of Bacillus subtilis natto was enhanced by 68% when hydroxylamine hydrochloride and UV mutagenesis were combined [43]. Another study investigated that the UV mutation breeding of *Bacillus subtilis* natto in order to enhance the production of Nattokinase and the strain yielded 534.95 U/mL of enzyme, which was 11.84% higher than that of the original strain [44]. On the other hand, Wang et al., mutated the wild-type strain *Bacillus subtilis* JNFE0126 using a combination of UV and 60Co-γ irradiation, producing a mutant strain (*Bacillus subtilis* JNFE1126) that yielded doubled Nattokinase production [45]. In our study *Bacillus subtilis* VITMS2 yielded 1034.18 93.1 ± 0.5 FU/mL and 1240 ± 1.09 FU/mL of enzyme production via physical and chemical mutation. The EMS mutagenesis effectively improved the NK production in *Bacillus subtilis* VITMS2, with EMSBS-14 showing approximately a threefold higher fibrinolytic activity compared to the wild-type. Similarly compared to the previous studies mentioned, the mutagenized VITMS2 strain achieved an approximately 2-to-4-fold higher Nattokinase production, highlighting its superior potential following mutagenesis alone. This improvement highlights the effectiveness of our mutagenesis strategy in generating high-yielding strains.

In this study, the mutagen treated EMSBS-14 isolate on optimization using response surface methodology and medium supplemented with agro-residues to enhance the fibrinolytic enzyme production. Agricultural residues and industrial process wastes offer a better alternative to nitrogenous substrates and also aid in the reduction of production costs. Similar strategies employing wheat bran, shrimp shell powder, cow dung, and soy by-products have been previously reported to improve fibrinolytic enzyme yields [12, 46, 47]. According to another study, the mutant strains EMS and EB-15 were reported to produce 2-4-fold fibrinolytic enzyme than the wild *B.cereus* GD 55 strain. On a general note, the use of solid substrate fermentation medium has been attributed to increase the production of enzymes [48]. Fibrinolytic enzyme produced by *Bacillus subtilis* WR350 on single- factor and orthogonal optimization to produce low-cost fermentation medium. The highest production of 5865 U/mL was achieved with the media components sucrose 35.0 g/L, corn steep liquor 20.0 g/L and MgSO4 2.0 g/L [49]. A study showed that nattokinase (NK) production has enhanced by 4.2-fold from *B. subtilis* A26 on using hulled wheat grains as substrate [50]. Powdered shrimp shell has been observed to increase the enzyme production in *Bacillus* s p. M2 by 2.32-fold. The strain *B. subtilis* IMR-NK has increased the yield of fibrinolytic enzyme up to 9.2-fold with a specific activity of 4400 U/mg when wheat bran was used a raw material [51]. In a study conducted the strain *Bacillus* sp. IND 7 demonstrated enhanced fibrinolytic activity with cow dung as substrate, it yielded 8,345 U/g substrate and overall production was improved to 2.5-fold [47]. Soy bean curd residue was used as a substrate for the Nattokinase production and the strain yielded 0.415 g/150 g of Nattokinase. Th strain also exhibited 1.3 FU/mL of fibrinolytic activity [52]. A study showed that *Bacillus subtilis* produced 2503.4 IU of Nattokinase when soy flour and rice husk were used as the medium components [53]. Another study optimized ideal fermentation conditions for the production of Nattokinase using a combination of OFAT, Plackett-Burman design, Box-Behnken design and the strain *Bacillus subtilis* yielded 7067.12 IU/g of enzyme [54]. A study reported that streptokinase produced from *Streptococcus equisimilis* exhibited an activity of 1200 U/mL [55]. According to the WHO, the standard values of commercially available thrombolytic agents such as streptokinase [56] and urokinase [57] were identified as 1013 IU/ampoule and 3200 IU/ampoule. When compared with the previous studies the strain isolated from fermented *Vigna unguiculata* milk yielded 4639.43 ± 10.65 FU/mL of Nattokinase which is quite higher. It also represents an approximate 2- to 9-fold enhancement compared to various previously reported studies on Nattokinase production. The enhanced production of Nattokinase was achieved through a combination of random mutagenesis (UV and EMS treatment) and statistical optimization with agro-residual substrates. These substrates not only improve enzyme production but also contribute to cost-effective and sustainable large-scale fermentation. The HPLC chromatogram exhibited a single peak with a retention time of 10.356 min which was comparable to nattokinase produced by *Bacillus* sp. SFN with a retention time of 10.633 min [36]. The molecular weight of the Nattokinase was found to be 29 kDa [25]. Hence, the sensitive and quick MALDI-Tof spectrometry revealed the purity and homogeneity with a single peak confirming the molecular mass of 29 kDa. Furthermore, the purified NK enzyme’s stability and fibrinolytic activity indicate its potential for therapeutic uses. Because of its higher yield, improved enzyme stability, and effective use of substitute substrates, *B. subtilis* VITMS2 is considered as a potential candidate for industrial NK production.

## Conclusion

In this work, the enzyme production was improved through systemic mutagenesis using random mutation of *B. subtilis* VITMS 2 with UV irradiation and treatment of ethyl methane sulfonate along with a lab-scale statistical optimization with agro-industrial wastes using response surface methodology. Notably, the use of agro-industrial wastes such as cane molasses, soybean waste, eggshell powder, and brewer’s spent grain as alternative carbon and nitrogen sources contributed to a substantial reduction in media cost, making the process economically viable. The increased enzyme yield achieved through these low-cost substrates and optimization strategies demonstrates that this approach is not only efficient but also cost-effective for potential scale-up. However, the study was limited to random mutagenesis approaches and lab-scale optimization; further research involving site-directed mutagenesis, gene expression studies, and molecular modifications using computer-aided modeling could help achieve even greater yields and process efficiency. The findings of this work highlight a promising, cost-effective strategy for the industrial production of Nattokinase, with opportunities for further enhancement through advanced molecular techniques.

## Data Availability

The strain used in this study was submitted to NCBI and the GenBank accession number is available from https://www.ncbi.nlm.nih.gov/nuccore/MK156734.1/.

## Abbreviations

UV: Ultraviolet
EMS: Ethyl methane solfonate
FU: Fibrinolytic units
NK: Nattokinase
HPLC: High performance liquid chromatography
MALDI-TOF: Matrix-Assisted Laser Desorption/Ionization Time-of-Flight
SDS-PAGE: Sodium dodecyl sulfate-polyacrylamide gel electrophoresis
RSM: Response surface methodology
CCD: Central composite design
